# Characterizing Cross-Provincial High-Cost Patients in Rural China: Cross-Sectional Study

**DOI:** 10.2196/54234

**Published:** 2025-06-11

**Authors:** Minjiang Guo, Xiaotong Jiang, Yang Liu, Yang Liu, Fangyuan Zhang, Zhongyuan Zhang, Yazi Li

**Affiliations:** 1 Health Insurance Information Research Department, Institute of Medical Information, Chinese Academy of Medical Sciences, Beijing, China; 2 Information Technology Office, Chinese Academy of Medical Sciences & Peking Union Medical College, No. 9 Dongdan Santiao, Dongcheng District, Beijing, 100730, China, 86 13810084409

**Keywords:** high-cost patients, cross-provincial, rural patients, China, inpatient

## Abstract

**Background:**

High-cost (HC) patients, typically defined as the top 10% or 5% of patients with the highest health care costs, are responsible for over half of all health care–related spending. In China, approximately 95% of rural residents are covered by Urban and Rural Resident Basic Medical Insurance. In parallel, increasing population mobility has made it more common for rural residents to seek medical treatment and claim reimbursements across provincial boundaries. These trends underscore the importance of identifying and understanding HC patients within this group.

**Objective:**

This study aimed to analyze the characteristics and risk factors associated with HC cross-provincial insured patients in rural China.

**Methods:**

The study used data from the cross-provincial medical immediate reimbursement system, which contains records of inpatients who used cross-province immediate reimbursement services between 2017 and 2019. Patients whose total annual medical expenditure ranked within the top 10% of all cross-provincial inpatients were classified as HC patients. Andersen’s Behavioral Model of Health Services Use was adopted to examine the factors associated with being an HC patient. Descriptive statistics and multivariable logistic regression model analyses were performed.

**Results:**

A total of 2987 patients were included, with a mean age of 42.99 (SD 19.39) years. Males comprised 57.4% (1713/2987) of the total. Among all cross-provincial patients, the expenses of HC patients made up 34.5% of total expenses. The average annual hospitalization cost per HC patient was US $22,460. Results from multivariable logistic regression analysis indicated that male patients (odds ratio [OR] 1.38, 95% CI 1.06-1.79; *P*=.01), individuals with multiple comorbidities (OR 3.62, 95% CI 2.37-5.53; *P*<.001), those diagnosed with cancer (OR 2.31, 95% CI 1.61-3.31; *P*<.001), and patients receiving care at specialized hospitals (OR 1.61, 95% CI 1.24-2.08; *P*<.001) were significantly associated with HC status.

**Conclusions:**

Cross-provincial HC patients in rural China exhibited a lower concentration of total expenditure but incurred higher average annual hospitalization costs compared with local patients. This finding suggests the presence of potential cost-driving factors within this group. Identified risk factors—including sex, comorbidity status, cancer diagnosis, and hospital type—may inform the development of more equitable and efficient health financing policies, such as optimizing resource allocation and designing targeted interventions for HC patients.

## Introduction

By 2010, China had achieved near-universal health coverage [1], and the population covered by the basic medical insurance (BMI) system has remained above 95% since 2011 [2]. The covered population reached 1.35 billion by the end of 2022 through 2 schemes [3], Urban Employee Basic Medical Insurance for formally employed workers and Urban and Rural Resident Basic Medical Insurance (URRBMI) for unemployed residents. However, due to the constantly deepening aging population and the deficiencies in the tiered health care system, BMI has been facing ever-increasing financial pressures in recent years [4]. The total expenditure of the BMI fund reached US $390 billion [5] in 2023, increasing 14.4% compared with 2022 [6], almost twice the growth rate of annual revenue (7.9%).

Along with the increasing trend, a unique but nonnegligible subgroup population who are facing substantial health care expense pressure is domestic migrants [7]. Due to the unbalanced development states across different regions of China, domestic migration has been increasing over the past 20 years, accounting for one-fourth of the Chinese population in 2020 [8], with 66.2% (249/376 million) being rural-to-urban migrants [9]. As the common migration route is from undeveloped areas to developed ones, it will be inevitable to trigger the increase of the health care cost for them. Besides, unbalanced distribution of health care resources usually accompanies the economy, which would be another “pull” for cross-regional health care and lead to higher health care expenses again. In 2021, China’s cross-provincial patients’ average hospitalization expenses were 2.17 times those of local-insured patients [10]. The rapidly rising medical costs have not only become a heavy burden for the migrant population but also pose great pressure on the medical insurance funds they participate in.

Identifying the characteristics of high-cost (HC) patients could help clearly establish priority actions for policy makers in the context of allocation of scarce resources and provide effective solutions to these challenges [11]. HC patients, usually defined as the top 10% [12] or 5% [4] of patients with the highest health care costs, are responsible for more than half of all health care–related spending [13,14]. Previous studies have examined the characteristics of HC patients, findings that 3 factors may be associated with HC patients [15]. The first is demographic characteristics, like age [16,17], gender [18,19], ethnicity [20,21], region [22,23], and so on. HC patients were generally older, but there is no consensus on gender or ethnic categories. The second is the financial status, including income and health insurance statuses. Several studies [24,25] have indicated that the cost reduction provided by insurance for patients is associated with an increased likelihood of beneficiaries becoming HC patients. Income was not significantly associated with high health care costs. In high-income countries, such as the United States, individuals with higher incomes are more likely to become HC patients, potentially due to their greater purchasing power and access to services [26]. In contrast, in low- and middle-income countries, such as China, individuals with lower incomes are more prone to severe illnesses, which increases their likelihood of incurring high health care costs [27]. The third is medical characteristics. The prevalence of chronic diseases and multimorbidity were dominant among HC patients [16,28,29]. Similar findings have been reported in studies conducted in China. For instance, research conducted in rural Dangyang City revealed that HC patients were older, more likely to belong to economically disadvantaged families, and exhibited a higher prevalence of chronic conditions, including tumors, chronic obstructive pulmonary disease, and cardiovascular diseases [27]. Another study conducted in rural Macheng City reported that female residents (12,320/23,922, 51.5%) and individuals aged more than 60 years (8240/23,922, 34.5%) with diseases that are challenging to diagnose exhibited a higher propensity to incur substantial medical costs [4].

Migration and displacement are significant factors that affect the health of individuals and hinder the achievement of health-related sustainable development goals. Addressing the health care needs of the migration population is of great significance in China. However, little is known about the characteristics of HC patients among domestic migrants. Analyzing the characteristics of HC patients within this population will offer critical insights for developing an inclusive health care system in their host cities. Meanwhile, from the perspective of patients, cross-regional medical treatment typically arises when the health care resources in their origin cities are inadequate to meet their medical needs, it will reflect deficiencies of health care resources of their flow-out cities, thereby facilitating more informed guidance on the allocation and utilization of health services. Compared with urban residents, rural-urban migrants faced greater financial constraints, and expected to be more vulnerable when using health care services.

This study aimed to examine the characteristics and risk factors associated with HC cross-provincial insured patients from rural China. First, we analyze the medical cost agglomeration characteristics of the rural-to-urban migrants. Second, we analyze the demographic, socioeconomic, health care utilization, and clinical characteristics of HC patients and non–high-cost (NHC) patients. Third, we adopt Andersen’s Behavioral Model of Health Services Use (AMHSM) as a theoretical framework (refer to Methods section) to identify the factors associated with HC patients among this population.

## Methods

### Design and Setting

We conducted a cross-sectional study using claims data from Hainan province. Hainan has a large outflow population. The seventh national census data from 2021 showed nearly 10% of Hainan’s locals live in other provinces [30], higher than the national average [31] (124,837,153/1,411,778,724, 8.8%). There are 2 possible reasons for this phenomenon. First, Hainan borders Guangdong, which is considered China’s economic powerhouse. Consequently, a significant number of the rural labor force from Hainan is inevitably drawn to Guangdong Province for employment, leading to increased demand for cross-province medical reimbursements. Second, medical resources in Hainan Province are relatively weak. According to the 2022 Health Statistics Yearbook [30], the number of licensed physicians in Hainan Province per 1000 people ranks 27th in China. The number of beds in medical institutions per 1000 population ranks 24th. With improvements in transportation and the increasing accessibility of medical insurance reimbursement, an increasing number of local patients travel to other provinces for better health care.

### Theoretical Model

AMHSM is the best theoretical model for studying health service utilization behavior [32]. In this research, we applied the AMHSM to examine the utilization of health services and the factors influencing it among the HC patients. This framework was validated and used in previous work on HC patients [33]. According to AMHSM, health services use was determined by (1) existing characteristics that predispose people to use or not use services even though these characteristics are not directly responsible for the use, like age, gender, and so on; (2) enabling characteristics that facilitate or impede the use of services, like income, insurance, and so on; and (3) need or condition, that refers to the level and type of experienced illness [15]. Furthermore, Anderson’s 2007 study [32] suggested that, in addition to individual factors, environmental factors should also be considered. In previous research, environmental factors were not considered primarily because the studied populations were situated within the same health care system environment. However, in the context of this study, the provinces and health care institutions into which the migrant population moves exhibit distinct environmental characteristics that may influence the level of health care utilization. Therefore, these factors were incorporated into the analysis.

### Data and Variables

All patients who utilized cross-provincial inpatient services and received immediate reimbursement were included. We collected data from Cross-provincial Medical Immediate Reimbursement System, which is a subsystem of URRBMI. The system was established in 2017, and it records the information related to cross-provincial medical reimbursement, including demographic characteristics, expenditure information, and clinical characteristics, among other variables (Table 1).

**Table 1.** Key variables in cross-provincial medical immediate reimbursement system.

Variables	Definition
Identification code	A unique identifier for an anonymized patient.
Sex	Male or female.
Age	Patient’s age in years.
Migration type	Migration type is a categorical variable, including illness-related referrals, working in other provinces, and living in other provinces.
Flow out city	The city (country or district level) where cross-provincial patients originate.
Flow in province	The province to which cross-provincial patients migrate for health care services.
Flow in hospital	The hospital where the cross-provincial patients received treatment.
Length of stay	The duration of hospitalization, calculated as discharge day minus admission day.
Medical cost per visit	Total medical costs for a single patient visit.
Reimbursed expenses per visit	Expenses reimbursed by health insurance per patient visit.
Out-of-pocket expenses per visit	Out-of-pocket costs per patient visit.
Diagnosis code	Coding according to *ICD-10^a^* system. Only primary diagnoses were recorded.
Diagnosis name	Naming according to *ICD-10* system. Only primary diagnoses were recorded.

a *ICD-10*: *International Classification of Diseases, Tenth Revision*.

### Independent Variables

Variables were categorized according to the AMHSM as follows. First, demographic characteristics, including age and gender. In addition, for the migrant population, we included the migration type as an independent variable. In China, patients seeking medical care outside their place of residence can generally be categorized into 2 types [34]. One group consists of individuals who have relocated to another province for occupational or personal reasons and subsequently required medical care. We defined them as migration-driven cross-province patients. The second group comprises individuals who sought better medical care outside their home province. We classify them as disease-driven cross-province patients. Logically, different types of patients exhibit distinct characteristics in terms of medical service utilization. Second, enabling characteristics, including income and insurance status. As the database did not include individual patient income information, the per capita disposable income of the patient’s insured county or city was used as a proxy. Regarding insurance status, like most studies using health insurance databases as data sources, this study included only insured patients, making it difficult to analyze whether insurance status is a predictor of HC patients. Third, need characteristics, including the diagnosis of disease and the number of diseases. The diagnosis was identified based on the *International Classification of Diseases, Tenth Revision* system [35]. The number of diseases refers to the number of primary diagnoses confirmed for a patient within a calendar year. Fourth, environmental characteristics, including regions and type of medical institutions where patients receive medical reimbursements. Regions were grouped according to the administrative provinces of China, and the top four provinces with the highest number of medical visits were listed separately, whereas the remaining provinces were uniformly classified as other areas. According to the *“*Law of the People’s Republic of China on Basic Medical and Health Care and the Promotion of Health”, health care institutions are classified into general hospitals and specialized hospitals. The variables and their assignments are illustrated in Table 2.

**Table 2.** Independent variables.

Variables		Code or values
Demographic characteristics		
	Age	Years
	Sex	Male=1, female=0
	Type of patients	Migration-driven cross-province patient=1, disease-driven cross-province patient=0
Enabling characteristics		
	Income	Continuous variables
Need characteristics		
	Number of diseases	Discrete variables
	History of diagnosis	yes=1, no=0
Environmental characteristics		
	Regions	Guangdong, Guangxi, Shanghai, Beijing, Other
	Medical institutions	Hospitals=0, specialized hospitals=1

### Dependent Variables

The dependent variable was a binary indicator, where a value of 1 represented that HC had been incurred. According to the report entitled “Effective Care for High-Need Patients: Opportunities for Improving Outcomes, Value, and Health,” published by the US National Academy of Medicine in 2017 [12], HC patients were defined as those whose total medical costs ranked among the top 10% of all cross-provincial patients within a calendar year. These costs included both out-of-pocket expenses and medical insurance reimbursement.

### Statistical Analysis

First, baseline descriptive statistics were calculated for HC patients, NHC patients, and overall patients. Counting data are expressed as relative numbers, and the chi-square test or Fisher exact probability method was used for intergroup comparisons. Measurement data conforming to a normal distribution are expressed as ($\overset{-}{x}$±s), and the least significant difference *t* test was used for intergroup comparisons. Measurement data not conforming to a normal distribution are expressed as median (IQR), and the Mann-Whitney *U* test was used for intergroup comparisons.

Second, we performed univariable and multivariable logistics regression analyses to identify the predictive risk factors of HC patients. Statistically significant variables (*P*<.05) in the univariable analysis were introduced in the multivariable model by using forward stepwise logistic regression. The odds ratio (OR) and 95% CI were calculated. All analyses were carried out using Stata 18 (StataCorp).

### Ethical Considerations

The study was approved by the Institutional Review Board of the Institute of Medical Information, Chinese Academy of Medical Sciences (IMICAMS/02/21/HREC). To safeguard patient privacy, personal information, including names and identification numbers, were blocked and excluded from the analysis. As the research involved secondary analysis of deidentified data, informed consent was exempted. No financial compensation was provided to either the patients or the researchers, and there were no funding sources that could create a conflict of interest for the study.

## Results

### Characteristics of Health Care Expenditure

**Figure 1.**
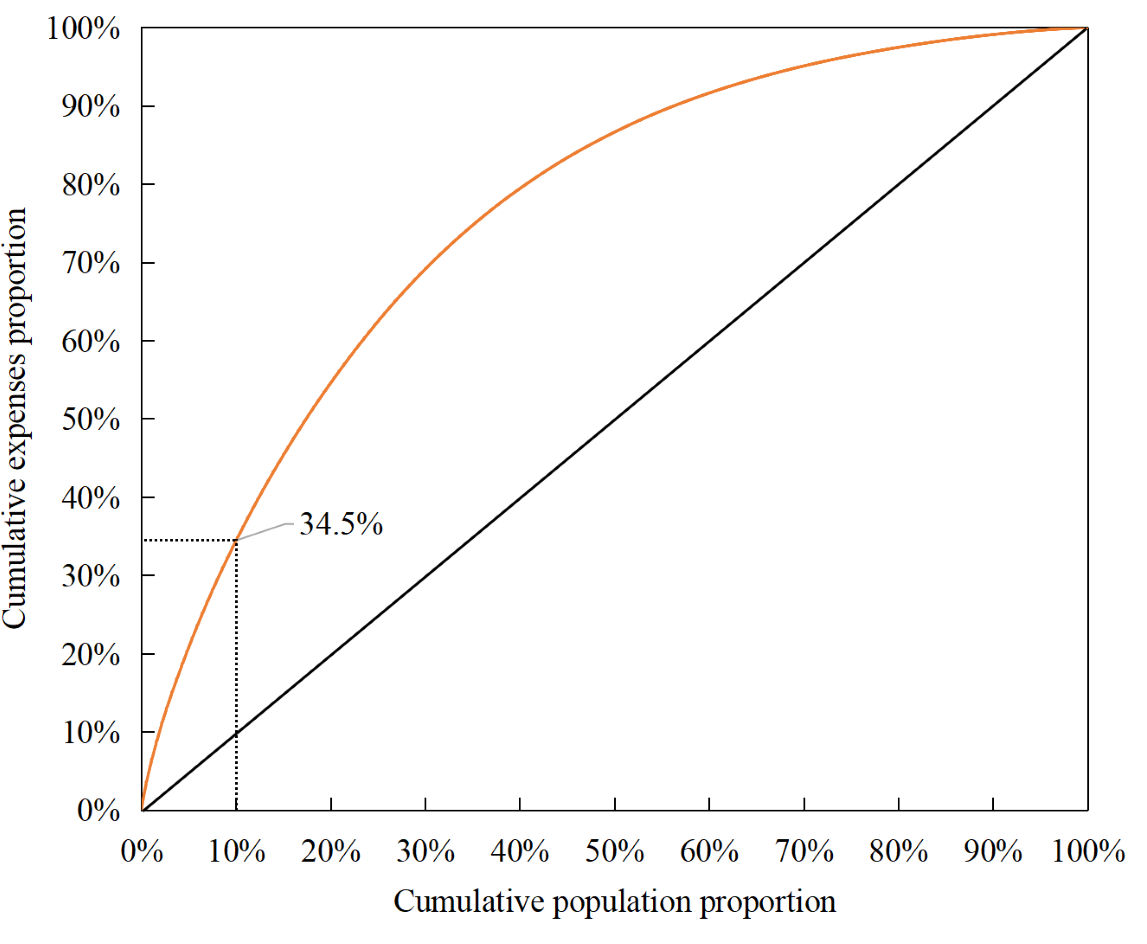
Overall distribution of annual medical expenditures referring to the cross-provincial patients.

Figure 1 illustrates the distribution of medical expenditures of cross-province rural patients. HC patients (299/2987, 10%) were responsible for 34.5% of total medical expenditures. The average cost per hospitalization for HC patients was US $6261, which was 2.47 times of the remaining patients. The disparity was even more pronounced in annual hospitalization costs, with HC patients incurring an average of US $22,460 per year, 6.34 times higher than NHC patients (Table 3).

**Table 3.** Comparison of hospitalization costs between high-cost and non–high-cost patients.

Costs	All patients (N=2987), median (IQR)	HC^a^ (n=299), median (IQR)	NHC^b^ (n=2688), median (IQR)	*P* value
Cost per hospitalization (in US $)	2852.10 (1406.07-5,753,51)	6260.86 (3999.69-17,942.18)	2531.65 (1319.81-5143.20)	<.001
Annual per capita hospitalization cost (in US $)	4301.25 (1678.82-10,520.53)	22,460.42 (19,439.39-27,140.60)	3545.40 (1551.54-8052.82)	<.001

a HC: high-cost.

b NHC: non–high-cost.

### Sample Characteristics

Table 4 illustrates the baseline characteristics for the sample. A total of 2987 patients were included in this study, with a median age of 47 (IQR 30.00-58.00) years. Males accounted for 57.4% (1713/2987) of total patients. Among all patients, migration-driven cross-province patients and disease-driven cross-province patients were evenly distributed, each comprising nearly 50% (1477/2987) of the total. The average number of diseases per patient was 1.06 (SD 0.27), and 48.4% (1446/2987) of the patients were diagnosed with neoplasms. Of these patients, 93.4% (2789/2987) went to Guangdong which had the highest gross domestic product in China in 2022 [36], which is geographically adjacent to Hainan Province. In addition, 63.7% (1904/2987) of these patients sought care in general hospitals.

**Table 4.** Characteristics of cross-provincial beneficiaries by cost subgroup.

Characteristic				All patients (n=2987)	HC^a^ (n=299)	NHC^b^ (n=2688)
Demographic characteristics						
	Age (years)					
		Median (IQR)		47.00 (30.00-58.00)	44.00 (31.00-56.00)	47.00 (30.00-58.00)
		≤18, n (%)		428 (14.3)	40 (13.4)	388 (14.4)
		18‐60, n (%)		1947 (65.2)	202 (67.6)	1745 (64.9)
		≥60, n (%)		612 (20.5)	57 (19.1)	555 (20.7)
	Sex, n (%)					
		Male		1713 (57.4)	196 (65.6)	1517 (56.4)
		Female		1274 (42.7）	103 (34.5）	1171 (43.6）
	Migration type, n (%)					
		Migration-driven cross-province patients		1477 (49.5)	88 (29.4)	1389 (51.7)
		Disease-driven cross-province patients		1510 (50.6)	211 (70.6)	1299 (48.3)
Enabling characteristic						
	Income (in RMB^c^), median (IQR)			13,374 (12,679-14,206)	13,374 (12,679-14,465)	13,374 (12,679-14,206)
Need characteristics						
	Number of diseases					
		Mean (SD)		1.06 (0.27)	1.17 (0.43)	1.05 (0.24)
		1, n (%)		2815 (94.2)	253 (84.6)	2562 (95.3)
		2, n (%)		159 (5.3)	40 (13.4)	119 (4.4)
		3, n (%)		10 (0.3)	6 (2.0)	4 (0.2)
		4, n (%)		3 (0.1)	0 (0)	3 (0.1)
	Top 10 diagnosis, n (%)					
		C00-D48 Neoplasms		1446 (48.4)	232 (77.6)	1214 (45.2)
		K00-K93 Diseases of the digestive		201 (6.7)	8 (2.7)	193 (7.2)
		M00-M99 Diseases of the musculoskeletal system and connective tissue		192 (6.4)	15 (5.0)	177 (6.6)
		N00-N99 Diseases of the genitourinary system		191 (6.4)	7 (2.3)	184 (6.9)
		I00-I99 Diseases of the circulatory system		167 (5.6)	22 (7.4)	145 (5.4)
		J00-J99 Diseases of the respiratory		128 (4.3)	8 (2.7)	120 (4.5)
		G00-G99 Diseases of the nervous system		111 (3.7)	5 (1.7)	106 (3.9)
		H00-H59 Eye and adnexa diseases		108 (3.6)	2 (0.7)	106 (3.9)
		Q00-Q99 Congenital malformations, deformations, and chromosomal abnormalities		100 (3.4)	7 (2.3)	93 (3.5)
		E00-E90 Endocrine, nutritional, and metabolic diseases		80 (2.7)	2 (0.7)	78 (2.9)
Environmental characteristics, n (%)						
	Regions					
		Guangdong		2789 (93.4)	289 (96.7)	2500 (93.0)
		Other area		106 (3.6)	3 (1.0)	103 (3.8)
		Beijing		41 (1.4)	4 (1.3)	37 (1.4)
		Guangxi		37 (1.2)	2 (0.7)	35 (1.3)
		Shanghai		14 (0.47)	1 (0.3)	13 (0.48)
	Medical institution type					
		Hospitals		1904 (63.7)	146 (48.8)	1758 (65.4)
		Specialized hospitals		1083 (36.3)	153 (51.2)	930 (34.6)

a HC: high-cost.

b NHC: non–high-cost.

c RMB ¥6.8985=US $1 in 2019.

Compared with NHC patients, the proportion of males was higher among HC patients (196/299, 65.6% vs 1517/2688, 56.4%), and the proportion of disease-driven cross-province patients among HC was higher than migration-driven cross-province patients. Regarding disease type, HC patients were more likely to suffer from neoplasms (C00-D48) and circulatory system diseases (I00-I99), whereas NHC group exhibited a higher prevalence of digestive diseases (K00-K93), genitourinary system disorders (N00-N99), nervous system diseases (G00-G99), eye and adnexa diseases (H00-H59), and endocrine, nutritional, and metabolic diseases (E00-E90). In addition, the proportion of HC patients with 2 or more diseases was higher than that among NHC patients.

### Risk Factors Associated With HC Patients

To identify the risk factors associated with HC status, 9 variables identified as significant in the univariable logistic regression analysis were included in the multivariable model. Male patients were more likely to be HC patients (OR 1.38, 95% CI 1.06-1.79; *P*=.01). The number of diseases (OR 3.62, 95% CI 2.37‐5.53; *P*<.001) and neoplasms (OR 2.31, 95% CI 1.61‐3.31; *P*<.001) remained strong predictors. In addition, receiving care at specialized medical institutions was positively associated with HC status (OR 1.61, 95% CI 1.24‐2.08; *P*<.001). However, migration type (OR 0.75, 95% CI 0.55‐1.04; *P*=.09) and some disease types, such as genitourinary disease, were no longer statistically significant after adjusting for covariates (Table 5).

**Table 5.** Risk factors associated with the high-cost patients.

Covariate (reference group)	Univariable analysis		Multivariable analysis	
	Odds ratio (95% CI)	*P* value	Odds ratio (95% CI)	*P* value
Sex (reference: female)	1.46 (1.14-1.89)	.04	1.38 (1.06-1.79)	.01
Migration type (reference: disease-driven cross-province patients)	0.39 (0.30-0.51)	<.001	0.75 (0.55-1.04)	.09
Number of diseases	2.95 (2.15-4.03)	<.001	3.62 (2.37-5.53)	<.001
Neoplasms (C00-D48)	4.19 (3.16-5.57)	<.001	2.31 (1.61-3.31)	<.001
Diseases of the digestive system (K00-K93)	0.35 (0.17-0.73)	.005	0.40 (0.18-0.90)	.03
Diseases of the genitourinary system (N00-N99)	0.33 (0.15-0.70)	.004	0.47 (0.20-1.09)	.08
Eye and adnexa diseases (H00-H59)	0.16 (0.04-0.67)	.01	0.19 (0.04-0.82)	.03
Endocrine, nutritional, and metabolic diseases (E00-E90)	3.01 (1.74-5.19)	<.001	0.83 (0.40-1.73)	.63
Types of medical institutions	1.98 (1.56-2.52)	<.001	1.61 (1.24-2.08)	<.001

The Hosmer-Lemeshow goodness-of-fit test yielded *χ*²_8_=3.5 (*P*=.90), indicating a good model fit.

We conducted the Hosmer-Lemeshow goodness-of-fit test to assess the adequacy of the logistic regression model. The results indicate a good model fits.

## Discussion

### Principal Findings

This study is the first to analyze the characteristics and risk factors of HC cross-provincial patients from rural China. The analysis of cost distribution revealed that, unlike local patients, whose medical expenses are nearly half-concentrated among HC patients, the expenditures of cross-provincial patients are more dispersed, accounting for 34.5% of total medical costs. In addition, our findings revealed that both the per-hospitalization and annual inpatient expenditures for cross-provincial patients were higher than those for local patients. This reflects the differences in cost structure and expenditure levels between local and cross-provincial patients.

We adopt AMHSM as a theoretical framework to identify the factors associated with HC patients. Unlike previous studies, we considered the environmental factors and migration type, reflecting the specific characteristics of cross-provincial patients. The results showed that in addition to gender, number of illnesses, and type of illness, the type of institution was also important predictor of HC patients. This finding can provide more precise evidence support for relevant policy making.

### Comparison With Previous Work

For policy makers, focusing on HC patients may be highly efficacious for reducing healthcare expenses. We therefore analyzed the characteristics and risk factors of HC patients among cross-provincial beneficiaries in rural China, which has been generally neglected by other researchers.

First, our study found that the expenses of HC patients accounted for only 34.5% of total expenses. This is substantially different from the cost distribution of local inpatients. According to the study of Fan et al [37] and Xiaobo and Chuang [38], the local HC beneficiaries in rural China were responsible for approximately 50% of total inpatient costs. By contrast, the cost structure of cross-provincial patients appears less concentrated. This may be attributed to the types of institutions eligible for reimbursement and to patients’ health care–seeking behavior. In the initial phase of policy implementation, in order to alleviate the financial burden on patients with serious illnesses and to account for variations in institutional information technology capacity, tertiary medical institutions served as the main providers of cross-provincial direct settlement for medical expenses. According to statistical data, by 2018, 96.6% (2462/2548) of tertiary medical institutions nationwide had been included in the network of direct cross-provincial settlement for medical expenses, compared with 58.1% (5238/9017) and 25.5% (2758/10,831) of secondary and primary institutions, respectively [34,39,40]. Due to the greater certainty of reimbursement in tertiary institutions, combined with patient’s trust in the clinical capabilities of higher-level providers, 99% (2957/2987) of cross-provincial patients in Hainan chose to seek treatment at tertiary medical institutions. Compared with local residents, this disproportionate pattern of health care utilization has reduced the number of patients within the lower-cost segment, thereby lowering the overall concentration of cross-provincial health care service utilization.

Second, we found that HC migrant patients incurred higher annual medical expenses than their local patients. Specifically, the average annual hospitalization cost for HC migrant patients was US $22,460, whereas that for local HC patients ranged from US $4398 to US $11,516 [38,41,42]. This may be related to selective medical migration. Notably, 70.6% (211/299) of HC patients are disease-driven cross-province patients, many of whom suffer from severe and complex conditions. These individuals often seek care at regions with well-developed health care resources and typically exhibit a high willingness to pay. Consequently, their medical expenditures tend to exceed those of local patients. However, the possibility of underlying inefficiencies in the utilization of medical resources cannot be ruled out [43,44].

The results of the multivariable logistic regression model revealed the characteristics and risk factors of HC patients. Our findings reveal that gender, number of diseases, cancer diagnoses, and type of hospital are independent risk factors for becoming an HC patient, whereas age, migration type, income level, other disease types, and treatment region are not. These results differ from studies conducted on local patients. In research focusing on local populations [4,28], age is often associated with HC patients, with older age linked to a higher risk. However, our study found no significant association between age and HC status, which may reflect the structural complexity of the migrant population. Compared with local patients, migrants are primarily composed of 2 types—disease-driven cross-province patients and migration-driven cross-province patients. Disease-driven cross-province patients are likely to consist of middle-aged and young individuals who are more mobile and willing to pay for care, while migration-driven cross-province patients have a more diverse composition, including freelancers, older adult individuals and children migrating with family members, and working-age adults relocating for employment. Consequently, the association between age and HC patients may vary depending on the changing composition of the migrant population. In a study of local patients, previous studies [45,46] have reported inconsistent results regarding the influence of gender on HC patient status. In our research, male patients were more likely to become HC patients, potentially reflecting traditional societal norms in China, where men are more likely to take on roles involving external work. Several studies on the health care–seeking behavior of the migrant population have reached similar conclusions [47,48], often attributing this to the perception that men are the primary economic providers and constitute valuable human capital within the household. As a result, their health is prioritized, and greater medical resources are more likely to be allocated to them.

We found that patients with chronic conditions, such as cancer, and those with multiple comorbidities were more likely to be HC patients. This may be attributable to the relatively high resource consumption associated with the treatment of such conditions, which is consistent with the findings of other studies [49]. We also found that HC cross-provincial patients tend to be concentrated among those with cancer and gastrointestinal diseases. However, studies of local patients have identified additional predictors of HC status, including neurological disorders [50], kidney diseases [51], and diabetes [52], as well as predictors of HC status. This suggests that cancer may represent a relative weakness in the local health care capacity in Hainan, prompting patients to seek care outside the province. By contrast, other HC conditions appear to be adequately treated locally and, as such, do not emerge as significant predictors of cross-provincial HC utilization.

Institution type also serves as a risk factor for becoming an HC cross-provincial patient. Our study found that individuals receiving care at specialist hospitals were more likely to incur high medical expenses, which may be related to the fact that these institutions are better equipped to manage complex and severe conditions, and consequently involve greater resource consumption and higher costs.

Migration type does not appear to be a key predictor for becoming HC patients. However, based on migration-related characteristics, disease-driven cross-province patients would be expected to have a higher likelihood of becoming HC users. To examine this further, we conducted a subgroup analysis by year (Multimedia Appendix 1). The results indicate that in 2017, disease-driven cross-province patients were more likely to become HC patients (OR 0.45, 95% CI 0.22‐0.93; *P*=.03), whereas in 2018 (OR 0.89, 95% CI 0.55‐1.66; *P*=.96) to 2019 (OR 0.94, 95% CI 0.60‐1.45; *P*=.94), there was no significant difference between disease-driven cross-province patients and migration-driven cross-province patients in the likelihood of incurring high medical costs. On the one hand, this may be related to limited awareness of the cross-provincial medical settlement policy. In 2017, when the policy had just been introduced, migrants living outside their registered residence were often situated in an information gap and unaware that medical expenses incurred elsewhere could be directly reimbursed. A survey from Shanghai in 2017 found that 64.5% (129/200) of migrant patients were unfamiliar with the policy [53]. Based on a relatively lower economic capacity and unawareness of the reimbursement policy, the migrant population may still choose to return to their hometown for treatment when they get seriously ill, which leads to a lower health care cost for migration-driven cross-province patients. As policy awareness improved over time, an increasing number of migration-driven cross-province patients chose to access health care services locally, resulting in a gradual rise in the proportion of HC users within this group. On the other hand, this may be related to the fragmented pattern of care-seeking typically observed among disease-driven cross-province patients. These patients often undergo initial diagnosis and treatment within their insured region, and only seek care at higher-level institutions outside the insured area when treatment proves ineffective. After a certain therapeutic effect is obtained, they tend to return to the insured location for rehabilitation treatment. However, the data used in this study were only drawn from the database of the Cross-provincial Medical Immediate Reimbursement System and did not capture pre- and posttreatment costs incurred within the insured region, which are recorded in the URRBMI system. As a result, the annual health care expenditure of disease-driven cross-province patients did not appear higher than that of migration-driven cross-province patients. The combined effect of low policy awareness and disease-driven cross-province patients’ fragmented care patterns may explain why there is no significant difference between the 2 groups in the likelihood of becoming HC patients.

Our findings provide valuable insights to inform decision-making. From the perspective of health resource allocation and utilization, local governments regularly invest in developing and improving health systems. The results of this study can guide resource allocation priorities. For example, we identified neoplasms as the highest-risk diagnosis among HC patients. Under this premise, policy makers can prioritize improving local diagnostic and treatment capacities for neoplasms and reduce the outflow of cross-provincial patients in Hainan, especially for disease-driven cross-province patients. This move brings at least 3 benefits. The first is to reduce direct medical expenses. Table 4 shows that 96.6% (289/299) of HC patients go to Guangdong for treatment. The 2020 Yearbook of Health Statistics shows that the average cost per hospitalization in Guangdong is US $1771, while that in Hainan is US $1479 [34], which means that receiving treatment in Hainan will cost less. The second is saving indirect costs, including the cost of transportation and accommodation. Finally, from the perspective of medical insurance fund supervision, it is more beneficial to the regulation and charge of the health care fund in the local medical institutions. Yu and Lang [54] and Auster and Oaxaca [55] demonstrated that differences in payment management mechanisms contribute to a higher likelihood of overtreatment when seeking health care in other provinces. Thus, patients are likely to receive more standardized medical treatment within their insured regions compared with cross-provincial patients. This can reduce unnecessary expenditures for medical insurance funds and out-of-pocket costs for individuals.

### Limitations

This study has several limitations. First, patients who were reimbursed manually were not included [56]. Second, due to policy restrictions, outpatients receiving cross-provincial medical care were not eligible for reimbursement before 2021, so only inpatients were included. Third, the medical insurance database lacks individual-level income information. Hence, the per capita disposable income of the registered residence area was used as a proxy.

### Conclusions

This study examined the characteristics and risk factors of HC cross-provincial insured patients in rural China. Our findings indicate that, unlike local patients, HC patients among the cross-provincial group did not exhibit a high degree of expenditure concentration, yet their average annual health care spending was significantly higher. This suggests the existence of cost-driving factors specific to cross-provincial patients—such as restrictions on reimbursable institutions, a preference for higher-level facilities, and insufficient fund oversight—which may contribute to both greater cost dispersion and elevated per capita health expenditure.

Our research also found that male patients, individuals with multiple comorbidities, those diagnosed with cancer, and patients receiving care at specialized hospitals were more likely to be classified as HC patients. Understanding these characteristics can assist policy makers in regions of patient outflow to develop targeted interventions—such as strengthening prevention and management of HC conditions and promoting more equitable allocation of health care resources—to reduce the outflow of patients with severe illnesses.

## Supplementary material

10.2196/54234 Multimedia Appendix 1 Multivariable analysis of the association between migration type and high-cost status by year (reference group: disease-driven cross-province patients).

## Abbreviations

AMHSM: Andersen’s Behavioral Model of Health Services Use
BMI: basic medical insurance
HC: high-cost
NHC: non–high-cost
URRBMI: Urban and Rural Resident Basic Medical Insurance
