# Supplementary material for: Characterizing Cross-Provincial High-Cost Patients in Rural China: Cross-Sectional Study
Source: JMIR Public Health Surveill. 2025 Jun 11;11:e54234. doi: 10.2196/54234 (PMC12176241; doi:10.2196/54234)
Supplement: Multimedia Appendix 1 [file publichealth-v11-e54234-s001.docx]

Multivariable analysis of the association between migration type and high-cost status by year (reference group: disease-driven cross-province patients)

| **year** | **Covariate**  **(reference group)** | **Multivariate analysis** | | |
| --- | --- | --- | --- | --- |
|  |  | **Odds Ratio** | ***P*-value** | **95% CI for OR** |
| 2017 | Migration type  (ref: disease-driven cross-province patients) | 0.45 | .031* | 0.22-0.93 |
| 2018 |  | 0.96 | .89 | 0.55~1.66 |
| 2019 |  | 0.94 | .77 | 0.60~1.45 |

Other variables: Controlled
